# Improving Blood Transfusion Request Form Documentation: A Quality Improvement Project

**DOI:** 10.7759/cureus.68942

**Published:** 2024-09-08

**Authors:** Waddah Ahmed, Ahmed Mohamed, Abubakr Muhammed, Abdalmahmoud Asadig Kanan Ahmed, Sara Omer Mohamed Abdalla, Abdelrahman Elfatih Elsheikh Abdelrahim, Dina Hashim WahidEldin Osman, Galaleldin Mohamed Abdeljalil Mohamed, Alaa Mobarak Abdalla Elkhalifa, Omer Mohamed Ibrahim Hamad, Shimaa Abdallah Mukhtar Mohammed, Suaad Bashary Saeed Ali, Islam A. A. Mahmoud, Ayman Hassan Elsiddig Mohamed

**Affiliations:** 1 Department of General Internal Medicine, Ribat University Hospital, Khartoum, SDN; 2 Department of Orthopaedics and Trauma, Gezira Centre for Orthopaedic Surgery and Traumatology, Wad Madani, SDN; 3 Department of Surgery, Al Managil Teaching Hospital, Al Managil, SDN; 4 Department of Public Health, University of Gezira, Wad Madani, SDN; 5 Department of General Medicine, Prince Sultan Military Medical City, Riyadh, SAU; 6 Department of Paediatrics, Algadarif Teaching Hospital, Algadarif, SDN; 7 Department of Surgery, Omdurman Islamic University, Omdurman, SDN; 8 Department of Internal Medicine, Wad Madani Teaching Hospital, Wad Madani, SDN; 9 Department of Dermatology, King Salman Hospital, Riyadh, SAU; 10 Department of General Medicine, Alshogig Primary Health Care, Gizan, SAU; 11 Department of Internal Medicine, Aswan Teaching Hospital, Aswan, EGY

**Keywords:** blood bank, documentation completeness, mean completion rate, quality improvement project, transfusion request form

## Abstract

Background: The transfusion quality improvement project (QIP) serves as a valuable tool for assessing and educating individuals who request blood components.* *The World Health Organization (WHO) recommends that each institution utilize a blood transfusion request form to ensure the effective conveyance of patient information to the hospital's blood bank. This QIP aimed to implement a transfusion request form and measure compliance with its use.

Methods: A prospective study was conducted at Al Managil Teaching Hospital, Sudan, from May 1 to August 3, 2024, to address the lack of standardized transfusion request forms. The study included three cycles involving pre-intervention analysis, two phases of intervention with training sessions, and post-intervention evaluations. The interventions focused on developing and implementing a new transfusion request form, training clinical physicians, and reinforcing the form's use. Data from 100 randomly selected transfusion request forms were analyzed for completeness and adherence.

Results: The study showed significant improvements in the completeness of transfusion request forms across three cycles. In the first cycle, no data were collected, highlighting the absence of standardized forms. During the second cycle, with the introduction of the new form, the completion rates varied: some fields, such as patient information and clinical details, were fully completed in 50 cases (100%), while critical clinical parameters, such as current hemoglobin (Hb) and platelet (PLT) levels, were completed in only four requests (8%). By the third cycle, there was a substantial increase in completion rates across all domains. For example, patient information fields achieved 100% completion in 50 cases, and clinical parameters saw significant improvement, with current Hb and PLT levels documented in 48 cases (96%). The mean percentage completion increased from 68.1% in the second cycle to 97.9% in the third cycle, demonstrating the effectiveness of the interventions and training sessions. Minor decreases were observed in health insurance documentation and certain clinical details, indicating areas for further improvement.

Conclusion: The systematic implementation and iterative evaluation of transfusion request forms significantly enhanced documentation completeness.

## Introduction

In 2008, the World Health Organization (WHO) recommended that each institution utilize a blood transfusion request form to ensure the effective conveyance of patient information to the hospital blood bank [1]. The "serious hazards of transfusion" report, which was published in 2008, indicates that the majority of transfusion errors were the consequence of inadequate communication, which was caused by incomplete requisitions sent to the blood bank [2]. Later in 2012, the British Committee for Standards in Haematology (BCSH) released its guidelines and advised that organizations establish local policies to reduce the likelihood of transcription or misinterpretation errors in all forms of communication, including written, verbal, and electronic communication [3].

The guideline also recommended that transfusion requisition slips must include the following variables: patient core identifiers, current diagnosis, significant comorbidities, clear unambiguous reason for the request, type of component and the volume of units required, clinical special requirements (such as irradiated, washed, or leuko-depleted blood), time required, patient location, requestor name, and contact number [3].

Interactions between laboratories and healthcare professionals, such as physicians, and other individuals involved in the healthcare system primarily take place through the use of request forms as a means of two-way communication.

Transfusion audits are valuable instruments for assessing the effectiveness of a procedure and educating individuals who are seeking blood components. Frequently, clinicians disregard specific information on laboratory request forms and the overall thoroughness of these forms, potentially leading to medical errors [4,5]. Therefore, conducting audits guarantees the detection of omitted information that may result in treatment delays, increase the likelihood of errors, and trigger a transfusion reaction, as demonstrated by multiple studies [6]. Additionally, Hospital Transfusion Committees (HTCs) have been established in various countries to manage and monitor all aspects of blood product transfusions, including ensuring their appropriate use within individual healthcare institutions [7]. The WHO recommends that every hospital should establish a transfusion committee to enforce national policies and guidelines while overseeing the use of blood and blood products at the local level [8].

In Sudan, transfusion request forms are primarily completed by house officers, who are in the initial stage of their clinical practice. Without proper training and supervision, they are more susceptible to making errors and providing incomplete information on these forms. This lack of experience can lead to significant issues in the accuracy and quality of the documentation, potentially compromising patient care and safety.

Unfortunately, at Al Managil Teaching Hospital, a standardized transfusion request form was previously unavailable, as clinical physicians relied on manual documentation using blank paper forms. The primary concern was the potential for incomplete or incorrect information, which could lead to administering the wrong blood type. This can result in severe reactions, such as hemolytic transfusion reactions, which can be life-threatening.

To address these issues, our quality improvement project (QIP) aimed to create a local standard transfusion request form at our hospital. The introduction of this request form was anticipated to streamline the transfusion process, enhance accuracy, and mitigate the likelihood of errors. Additionally, this study evaluated the adherence of clinical physicians to the new local form and assessed its impact on the overall transfusion process. By measuring both compliance and effectiveness, we seek to improve the efficiency and safety of transfusion practices within the hospital.

## Materials and methods

This prospective study was conducted at the Al Managil Teaching Hospital, Sudan, a major facility serving a diverse population.

First cycle (pre-intervention state and the root cause analysis: May 1-June 1, 2024)

Before the intervention, the study identified a critical issue at Al Managil Teaching Hospital: the absence of standardized transfusion request forms. This lack of standardization led to inefficiencies and potential errors in the transfusion process. Observations and consultations with stakeholders, including the Blood Bank Department, consultants, and hospital managers, confirmed this as the root cause.

Intervention 1 (June 1-June 15, 2024)

The initial intervention involved developing and introducing a new local transfusion request form (Appendix) following a meeting with the aforementioned stakeholders. This process began with extensive collaboration with relevant departments to design a form that addressed the issue. Once the form was finalized, initial training sessions were conducted for clinical physicians. These sessions aimed to ensure that all physicians understood how to properly use the new form and recognized its importance in improving the transfusion process. The training covered the rationale behind the new form, detailed instructions on how to complete it, and the expected benefits of its use, such as reduced errors and increased efficiency. Additionally, we conducted focus group discussions and displayed posters. This comprehensive approach was designed to facilitate a smooth transition to the new system and to promote widespread adoption among the hospital staff.

Second cycle (post-intervention state: June 16-July 2, 2024)

After the first intervention, the study entered the post-intervention phase. This phase involved evaluating the effectiveness and adherence to the new standard through a detailed review of 50 randomly selected transfusion request forms to assess adherence and gather suggestions for improvement.

Intervention 2 (July 3-July 17, 2024)

The second intervention focused on reinforcing the use of the new form. This included additional training and adjustments to the form based on feedback and suggestions from the initial implementation. Additionally, we discussed with the laboratory staff the importance of not accepting the form unless it is fully completed.

Third cycle (post-intervention state: July 18-August 3, 2024)

Following the second intervention, another review of 50 randomly selected transfusion request forms was conducted to ensure continued compliance with the established standards and to evaluate improvements or new issues that emerged from the previous cycle.

Data analysis

Data collected from the reviews of transfusion request forms underwent rigorous analysis. This analysis aimed to assess completeness and adherence to the new local standard, and identify areas for further improvement.

Evaluation

The evaluation phase focused on assessing the impact of the interventions. It included reviewing feedback, identifying strengths and weaknesses in the new form's implementation, and gathering suggestions for future enhancements.

Ethical approval

Ethical approval was obtained from the Institutional Review Board at Al Managil Teaching Hospital (approval number SUD24/04/Apr-26). This ensured that the study complied with ethical standards, including anonymization of personal and clinical data to protect privacy and confidentiality during analysis.

## Results

Table 1 shows the data collected over three cycles regarding the completeness of transfusion request forms. The domains assessed include patient information, clinical details, and necessary consent. The results indicate significant improvements from the first cycle to the third cycle, with a notable increase in the percentage of completed fields.

**Table 1:** Progress in Documentation of Transfusion Request Forms Across Three Cycles N/A = not applicable. The data have been represented as numbers (N) and percentages (%).

Domain	First Cycle	Second Cycle, N (%)	Third Cycle, N (%)	Improvement, %
Patient’s Name	N/A	100 (100%)	50 (100%)	-
Patient’s Date of Birth	N/A	42 (84%)	50 (100%)	16
Patient’s Age	N/A	45 (90%)	50 (100%)	10
Health Insurance	N/A	40 (80%)	39 (78%)	-2
Unit Name	N/A	48 (96%)	100 (100%)	4
Department	N/A	100 (100%)	100 (100%)	-
File Number	N/A	15 (30%)	100 (100%)	70
Consultant	N/A	49 (98%)	100 (100%)	2
House Officer	N/A	48 (96%)	100 (100%)	4
Clinical Details/Procedure	N/A	100 (100%)	49 (98%)	-2
Sample Collection Details	N/A	49 (98%)	49 (98%)	-
Current Hemoglobin and Platelet Levels	N/A	4 (8%)	48 (96%)	88
Target Hemoglobin and Platelet Levels	N/A	3 (6%)	47 (94%)	88
Known Antibodies	N/A	0 (0%)	100 (100%)	100
Previous Anaphylaxis	N/A	0 (0%)	100 (100%)	100
Test Required	N/A	0 (90%)	100 (100%)	100
Fetal Details If Applicable	N/A	0 (0%)	100 (100%)	100
Blood Component Required	N/A	48 (96%)	100 (100%)	4
Quantity Required	N/A	45 (90%)	49 (98%)	8
Indication For Blood Transfusion	N/A	100 (100%)	48 (96%)	-4

In the first cycle, the data collection was non-existent, indicated by N/A across all domains. This signifies that no transfusion request forms were utilized, or the data were not recorded. This baseline emphasizes the need for introducing and implementing a standardized form to ensure all critical information is documented.

In the second cycle, the implementation of the transfusion request form began, with varying degrees of completeness across different domains. Patient information fields such as names and department details were recorded in 50 (100%) forms. The date of birth (D.O.B.) and age fields had completion rates of 42 (84%) and 45 (90%), respectively. Health insurance information was included in 40 (80%) forms. The unit’s name and house officer fields were documented 48 (96%) times. Clinical details and procedures were fully completed at 100 (100%), but critical fields like current hemoglobin (Hb) and platelet (PLT) levels were recorded only 4 (8%) times. Other important areas such as known antibodies, previous anaphylaxis, test requirements, and fetal details (if applicable) were not documented in any of the forms. Additionally, the target Hb and PLT levels were poorly documented, with completion rates of 3 (6%). Blood component and quantity required fields showed high completion rates of 48 (96%) and 45 (90%), respectively.

The third cycle shows a significant improvement in most domains, reflecting the positive impact of continuous form usage and possibly improved training and awareness. Patient information fields reached a completion rate of 50 (100%) across the board. However, the health insurance field saw a slight decrease to 39 (78%). Clinical details and procedures remained consistently high, though there was a slight drop to 49 (98%). Consent for sample collection remained at 49 (98%), while documentation of current Hb and PLT levels increased dramatically to 48 (96%). Target Hb and PLT levels also saw a significant improvement to 47 (94%). The recording of known antibodies, previous anaphylaxis, test requirements, and fetal details (if applicable) all reached a perfect completion rate of 50 (100%). Blood component and quantity required fields maintained high levels of completion at 50 (100%) and 49 (98%), respectively.

The percentage improvements highlight areas of significant progress. The file number field increased by 70% (15 to 100), indicating better tracking and identification of patient records. Current Hb and PLT levels and target Hb and PLT levels both saw an 88% increase, reflecting improved attention to critical clinical parameters. Known antibodies, previous anaphylaxis, test required, and fetal details all showed a 100% increase, demonstrating excellent progress in capturing essential medical information. Despite the overall positive trend, health insurance documentation saw a slight decrease of 2% (40 to 39), suggesting an area needing attention. Clinical details/procedure and indication for blood transfusion experienced minor decreases of 2% (49 to 48) and 4% (50 to 48), respectively, indicating a need for further consistency in documentation.

**Figure 1:**
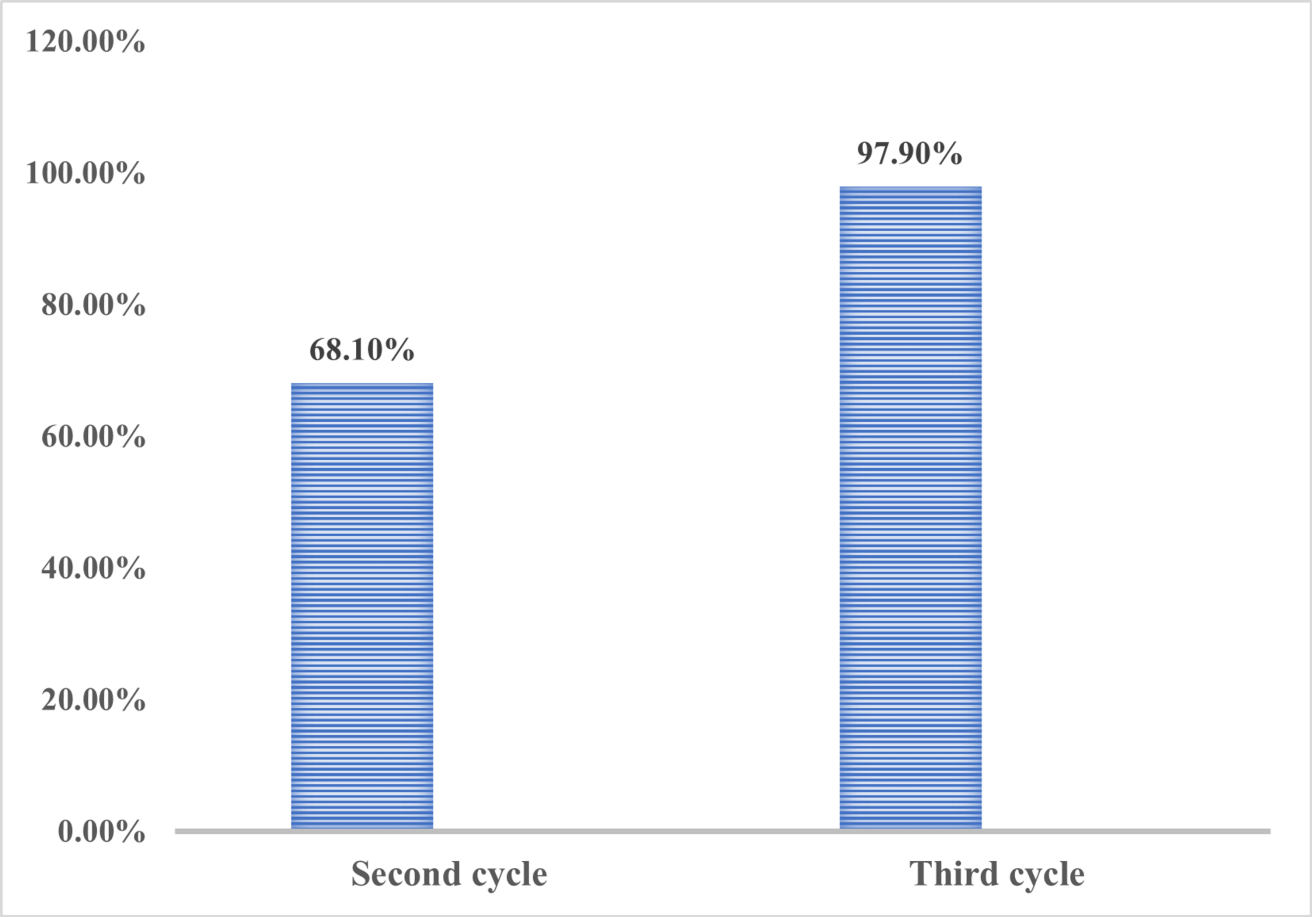
Mean compliance percentage in the second and third cycles The data have been represented as %.

The mean percentage of completion improved from 68.1% in the second cycle to 97.9% in the third cycle (Figure 1). This substantial progress underscores the effectiveness of iterative cycles in enhancing the completeness and accuracy of transfusion request forms.

## Discussion

Many healthcare providers mistakenly believe that they can request blood components without providing clinical details of the patient, based solely on a blood sample or blood group report [9]. Healthcare professionals who provide guidance and recommendations to patients regarding transfusion therapy have the duty to accurately depict the patient's information, as well as the specifics of the components involved [10].

The findings from the three cycles of evaluating the completeness of transfusion request forms demonstrate substantial improvements. We observed a dramatic shift from the absence of a standardized form to the implementation of a new one, resulting in nearly 100% completion of most parameters. This progression highlights the efficacy of iterative cycles and targeted interventions in enhancing form completion rates, thereby improving the overall quality of patient care.

In a study conducted by Jain et al. (2015) [11], it was discovered that 19.8% of blood request forms were not filled out completely. Similarly, Jegede et al. (2016) [12] found that 18.8% of forms lacked complete patient details.

Additionally, a study conducted over more than four months in Pakistan in 2017 analyzed 5,957 blood request forms and found that only 12.7% were fully completed by doctors requesting blood components at a tertiary care hospital [13]. In contrast, a subsequent study by Ghazanfer et al. [14], published in 2020 and conducted over six months at another tertiary care hospital in Pakistan, reported that only 6.8% of the forms were fully completed. In the current study, 31.90% of the blood requisition forms were found to be incomplete during cycle 2.

Comparing our study to that of Deb et al. (2001) [15], which found that 3.7% of transfusion request forms did not include the indication for transfusion, our results show a significant improvement. In the second cycle of our study, 100% of the forms included the indication. However, in the third cycle, this figure slightly decreased to 96%. Despite this small drop, the overall compliance rate remains notably high, reflecting the effectiveness of the implemented interventions and ongoing improvements in the completeness of transfusion request documentation.

Overall, the mean percentage completion improved from 68.1% in the second cycle to 97.9% in the third cycle, reflecting a substantial improvement of 29.8%. This progress highlights the effectiveness of the interventions implemented between cycles and underscores the value of continuous monitoring and feedback in enhancing clinical documentation practices.

Several limitations should be noted. The study was conducted at a single institution, which may not reflect practices or challenges in other healthcare settings. The short evaluation period may not capture long-term trends, or the sustainability of the improvements observed. These factors highlight the need for further research to ensure the generalizability and long-term effectiveness of the interventions.

## Conclusions

The systematic implementation and iterative evaluation of blood transfusion request forms have significantly enhanced the completeness of critical patient and clinical information documentation. While overall trends show improvement, ongoing efforts to address minor declines and emphasize thorough documentation are crucial to sustaining and further enhancing the efficiency of the blood transfusion request process. These findings provide a robust framework for institutions seeking to optimize their clinical documentation systems, thereby bolstering patient safety and elevating treatment standards.

## Figures and Tables

**Figure 2:**
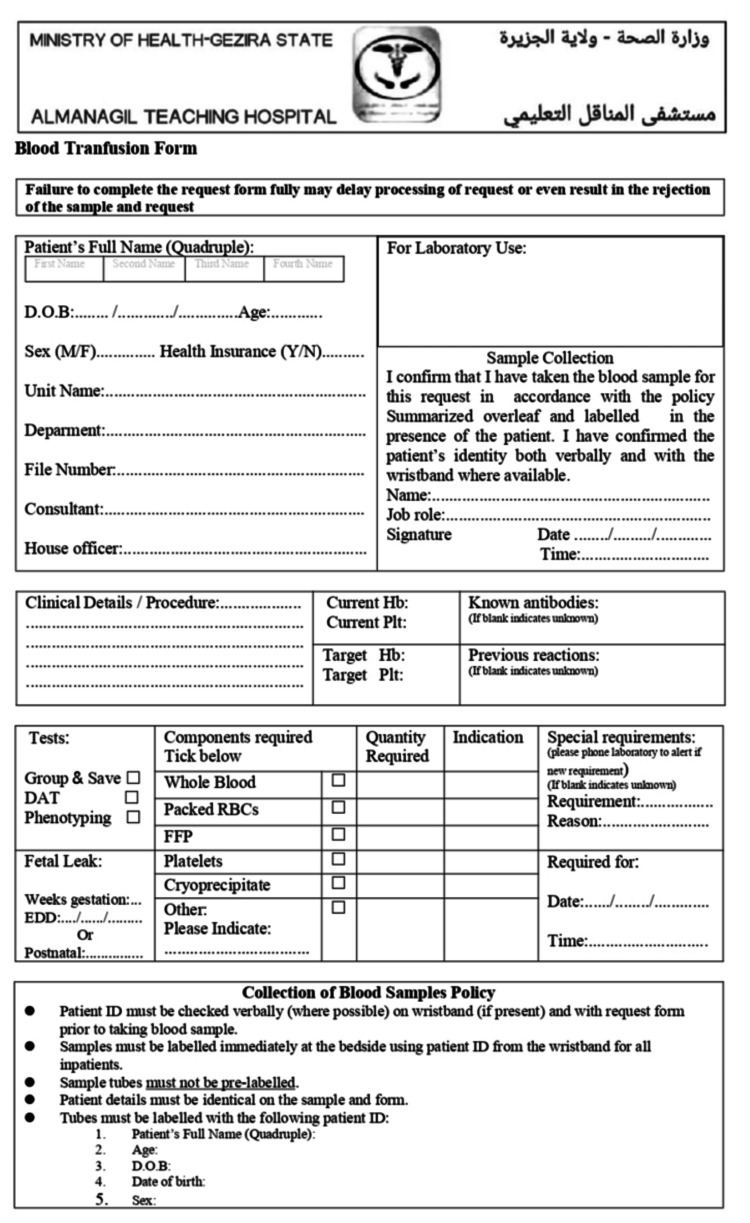
The new blood transfusion request form

## References

[1] Murphy MF, Stanworth SJ, Yazer M (2011). Transfusion practice and safety: Current status and possibilities for improvement. Vox Sang.

[2] Treleaven J, Gennery A, Marsh J (2011). Guidelines on the use of irradiated blood components prepared by the British Committee for Standards in Haematology blood transfusion task force. Br J Haematol.

[3] Harris AAC, Atterbury CLJ, Chaffe B (2012). Guideline on the Administration of Blood Components. https://b-s-h.org.uk/media/5152/admin_blood_components-BCSH-05012010.pdf.

[4] Bailey J, Jennings A, Parapia L (2005). Change of pathology request forms can reduce unwanted requests and tests. J Clin Pathol.

[5] Nutt L, Zemlin AE, Erasmus RT (2008). Incomplete laboratory request forms: The extent and impact on critical results at a tertiary hospital in South Africa. Ann Clin Biochem.

[6] Callum JL, Kaplan HS, Merkley LL (2001). Reporting of near-miss events for transfusion medicine: Improving transfusion safety. Transfusion.

[7] Haynes SL, Torella F (2004). The role of hospital transfusion committees in blood product conservation. Transfus Med Rev.

[8] World Health Organization. Blood Transfusion Safety Team (2001). The Clinical Use of Blood: Handbook. https://iris.who.int/handle/10665/42396.

[9] Pandey P, Setya D, Mirza SM, Singh MK (2021). Prospective audit of blood transfusion request forms and continuing medical education to optimise compliance of clinicians in a hospital setting. Transfus Med.

[10] Alving B, Alcorn K (1999). How to improve transfusion medicine. A treating physician's perspective. Arch Pathol Lab Med.

[11] Jain A, Kumari S, Marwaha N, Sharma RR (2015). The role of comprehensive check at the blood bank reception on blood requisitions in detecting potential transfusion errors. Indian J Hematol Blood Transfus.

[12] Jegede F, Mbah HA, Dakata A, Gwarzo DH, Abdulrahman SA, Kuliya-Gwarzo A (2016). Evaluating laboratory request forms submitted to haematology and blood transfusion departments at a hospital in Northwest Nigeria. Afr J Lab Med.

[13] Waheed U, Azmat M, Wazeer A, Sultan S, Irfan SM, Zaheer HA (2017). Evaluation of blood requisition and utilization practices at a tertiary care hospital blood bank in Islamabad, Pakistan. Glob J Transfus Med.

[14] Ghazanfer S, Aziz M, Mahmood HO, Rafi S (2020). Haemovigilance: Role and importance of blood request forms in routine hospital practice. Professional Med J.

[15] Deb P, Swarup D, Singh MM (2001). Audit of blood requisition. Med J Armed Forces India.

